# A Qualitative Exploration of Relations Between Salutogenesis and Persistent Music Performance Among Refugees Resettled in the United States

**DOI:** 10.3390/ijerph22010009

**Published:** 2024-12-25

**Authors:** Bernard Austin Kigunda Muriithi, Jennifer Muriithi

**Affiliations:** 1 Department of Communication Disorders and Occupational Therapy, College of Education and Health Professions, University of Arkansas, Fayetteville, AR 72701, USA; 2 College of Health Professions, University of Arkansas for Medical Sciences Northwest Regional Campus, Fayetteville, AR 72703, USA

**Keywords:** music, salutogenesis, sense of coherence, generalized resistance resources, refugees, health promotion, adaptation, trauma healing

## Abstract

In its broadest meaning, salutogenesis denotes an orientation toward the origins and assets for positive health, as opposed to the origins and risk factors associated with disease (i.e., pathogenesis). While salutogenesis continues to inspire health promotion, it has been noted that qualitative studies can further its understanding and broaden its use in research, clinical practice, and policies. The present study is based on an analysis of structured and unstructured recorded interviews with six refugee musicians. Applying interpretive phenomenological analysis, we comparatively explored the factors contributing to persistence in music between participants. Five factors were found: (a) long-term participation and identity as musicians; (b) desirable effects of music performance; (c) competence, talent, and social recognition; (d) management of social and cultural differences; and (e) locally organized refugee events. The links between music performance and salutogenesis include the following: (1) the inclusion of salutogenic orientation (in addition to pathogenic orientation) toward health; (2) that music invigorated generalized resistance resources so that their expression was augmented, steady, and enduring; and (3) that a high sense of coherence (SOC) was demonstrated. Future studies are needed to reveal whether other activities can take similar roles in developing SOC, and experimental studies are needed before health policy recommendations regarding activities can be made for people that need improved SOC.

## 1. Introduction

Since its introduction more than 40 years ago [1,2], salutogenesis has inspired research on health promotion, but qualitative research to increase understanding and expand perspectives has been limited [3]. Salutogenesis comprises the following: (1) salutogenic orientation (emphasizing health promotion rather than the cure or prevention of disease); (2) salutogenic model of health (SMH) and generalized resistance resources (GRRs), the factors associated with positive health; and (3) sense of coherence (SOC), the key construct representing what Antonovsky saw as a modifiable variable that predicts good health [1,2]. Salutogenic orientation emerged as a complement to pathogenic orientation, which is well established in healthcare practice [4]. To highlight differences between the paradigms, Antonovsky described salutogenic orientation as a perspective that is health-oriented rather than disease-oriented, a continuum (ease-dis/ease) rather than a dichotomy, as heterostatic rather than homeostatic, as holistic rather than specific, as proactive rather than reactive, and as oriented toward salutary factors (which promote health) rather than risk factors (which create disease) [1,5]. Further development of the salutogenic model of health (SMH), an improved understanding of SOC, the development of applicable assessment and interventions, and applications of salutogenesis beyond the health sector have been recommended [3].

The concept of GRRs, like salutogenic orientation, is presented as a continuum: Generalized Resistance Resources (GRRs) ↔ Generalized Resistance Deficits (GRDs) [5]. Antonovsky defined GRRs as “phenomena that provide one with sets of life experiences characterized by consistency, participation in shaping outcomes and an underload-overload balance” [5] (p. 19). GRRs include concepts such as material resources, knowledge and intelligence, ego identity, coping strategies, social support, commitment and cohesion with one’s culture, cultural stability, ritual activities, religion and philosophy, preventive health orientation, genetic and constitutional make up, and one’s state of mind [5]. GRRs and GRDs shape an individual’s SOC (a modifiable variable).

SOC is perhaps the most widely applied contribution in Antonovsky’s salutogenesis. It refers to “a global orientation that expresses the extent to which one has a pervasive, enduring though dynamic feeling of confidence that (1) the stimuli from one’s internal and external environments in the course of living are structured, predictable, and explicable; (2) the resources are available to one to meet the demands posed by these stimuli; and (3) these demands are challenges, worthy of investment and engagement” [5] (p. 19). Put differently, SOC is one’s own ability to identify and use internal and external resources to promote health [6]. It has been measured using the SOC scale (several versions).

As of 2019, more than 1700 studies have applied the SOC scale, revealing an association between SOC and good health [6], quality of life [7], and job stress [8], among other health-related outcomes. SOC has a strong relationship with positive attributes, such as resilience, hardiness, and coping [9]. It is considered a resource rather than a coping strategy and appears to protect against negative health (e.g., anxiety, depression, burnout, and hopelessness) [9]. High SOC can significantly support health for people experiencing adverse life events such as political violence, physical or sexual abuse, and war crimes. Therefore, understanding ways to promote SOC could serve to promote health for the more than 122 million forcefully displaced persons [10].

Studies on salutogenesis involving refugee participants have been mostly quantitative, focusing predominantly on evaluating associations between SOC and health and well-being outcomes [11]. Qualitative studies are limited in number and have referred to salutogenic approaches, but fewer have applied specific components of SMH like SOC or generalized resistance resources (GRRs). From these studies, spirituality and religion have emerged as an important GRR [11]. As exploratory, naturalistic, and interpretive, qualitative studies have the potential to deepen our understanding of the nature and working of GRRs, SOC, and salutogenesis in general. They could help address some criticisms of salutogenesis by broadening perspectives, specifying intervention tools or approaches, and clarifying some ways to conceptualize, apply, or test salutogenesis. Specifically, such studies could help researchers better understand the components of salutogenesis which are considered complex, vague, unclear, and difficult to empirically test [3]. One such outcome was a suggestion that SOC should include an understanding of how and why GRRs work [12]. Because it is recognized that activities or behaviors impact SOC [13], a greater understanding of how specific activities impact SOC could be beneficial in clinical intervention, research, and policy development.

In the current analysis, we aim to increase understanding of the relationship between one activity type (music performance) and salutogenesis. The initial publication arising from the same dataset described music as a tool for healing, or painkiller (i.e., it helped control distress and negative emotions), a conduit for communication and preservation of hope, and a means for social integration [14]. It was made clear in that article that participants held a predominantly pathogenic view (i.e., managing or preventing symptoms of disease), but they also, to a good extent, featured the salutogenic orientation (i.e., use of resources to promote health). Understanding associations between persistent music performance and salutogenesis required additional analysis of data. Relative to the persistence of music performance, our analysis was guided by the following questions: Why? What for? How so? We explore responses to these questions in our analysis and, in the discussion that follows, consider if and/or how the emerging factors contributing to persistence in music relate to salutogenic orientation, GRRs/GRDs, and SOC.

## 2. Methods

The study was approved by the A. T. Still University of Health Sciences’ Institutional Review Board (Protocol IRB #2019-015). Participants were informed of the aims, methods, risks, and benefits of the study and signed an informed consent document.

### 2.1. Research Design

The phenomenological design was selected because it allows researchers to provide a rich account of a small number of participants [15]. This design supports an exploratory approach to inquiry, applying inductive methods to describe or explain experiences that are unique to specified individuals. Like other qualitative designs, phenomenology does not aim to deductively prove or disprove the hypothesis. Instead, inquiry aims at increasing the understanding of complex experiences in which multiple realities may emerge [15,16]. The lived experience of refugee musicians who come from different countries and share diverse values is a complex reality that fits this design. A small sample allows sufficient comparisons of experiences between participants [15].

Phenomenology eschews the use of interpretive frameworks in the quest for comprehension of reality, thus necessitating the practice of bracketing (epoche) [15,17]. This requires the researcher to refrain from initiating the study with preconceived concepts and frameworks—such as SMH, SOC, or GRRs—which serve as a basis for comparing or contrasting ideas with the new data collected. Our methodology involved the application of descriptive phenomenology in the analysis section, deliberately excluding the influence of salutogenesis. Findings from this stage of analysis were presented at a conference [18]. Subsequently, within the discussion section, we identify and elucidate the connections between the emergent factors and salutogenesis.

### 2.2. Study Participants

Purposive and snowball sampling methods were used to identify six participants (see Table 1), as advised for studies using this research design [15,17]. The six participants, consisting of four individuals from the Democratic Republic of Congo, one from Burundi, and one from Myanmar, engaged in musical performances within a major metropolitan area of the United States. Four of these individuals resided in the city itself, while the other two resided in an adjacent city. At the time of the interview, the musicians had a history of participating in musical performances in the United States spanning two to ten years. Four of the musicians had recently resettled in the United States, with an approximate duration of two years since their arrival. All participants were employed in other sectors and did not consider music their main source of income. Among the Congolese musicians, there was a duo consisting of a mother and a son who regularly collaborated in performances. All Congolese musicians had resided in Uganda before their relocation to the United States. The Burundian musician had lived in a refugee camp in Tanzania prior to being resettled in the United States as a teenager. The sole participant who was resettled from Asia also served as a pastor for a Christian congregation composed of Asian immigrants and their families who gathered for worship each Sunday under his guidance.

### 2.3. Data Collection Procedures

**Figure 1:**
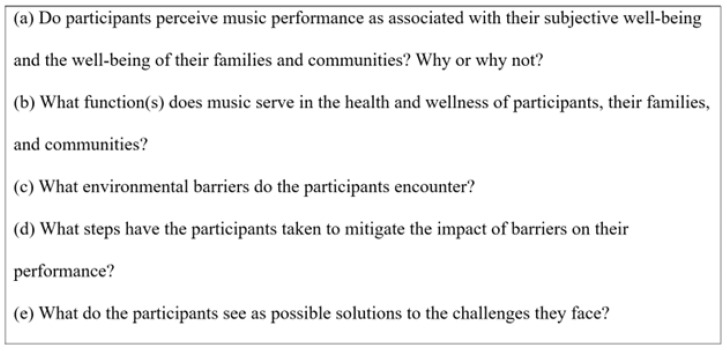
Interview guide.

In phenomenological research methodologies, the use of structured and unstructured interviewing techniques is endorsed [15,17]. Open-ended queries were used with flexibility to facilitate a conversational interview format, while supplementary questions were introduced, as needed, to elicit more comprehensive and detailed responses [17]. A 5-item questionnaire, comprising open-ended questions, guided all structured interviews (Figure 1). Each structured interview session with a participant was followed by an unstructured interview, with the entire process being recorded and later transcribed. Although certain participants were observed during their performances at community events, the observational data derived from these activities are excluded from the present analysis.

The initial interviews were conducted at the residences of study participants in English or Kiswahili. An in-depth interview was estimated to require about 75 min; however, participants were allowed to extend this time to ensure that their full story had been told. Short subsequent unstructured interviews of up to 10 min, either in person or by telephone, were additionally conducted to address questions arising during initial analysis to add depth and for member-checking. These later conversations were not audio-recorded.

### 2.4. Data Analysis Procedures

Interpretive phenomenological analysis (IPA) was employed with the aim of thoroughly exploring and describing the experiences of these individuals. IPA is characterized as iterative, flexible, dynamic, and multidirectional, which can result in outcomes that are creative, insightful, and potentially novel [15]. Finlay [17] describes the IPA method as comprising three fundamental components: an emphasis on subjective accounts of individual experiences, a commitment to comprehending experiences from the participants’ perspective, and an adherence to the hermeneutic approach. The process begins with the collection of idiographic accounts of individual experiences and concludes with the researcher’s synthesis of these accounts to articulate the collective experiences of a phenomenon.

Our initial analysis phase involved examining the data to identify individual perspectives that provided justifications for the persistence of music performance within the given context. This was followed by triangulation to identify commonalities among these perspectives. Follow-up interviews were conducted as needed to check the accuracy of reports and deepen the analysis. This methodological approach allowed the identification of factors expressed by all participants as contributing to the persistence of music performance.

## 3. Results

On the questions why, what for, and how so, the lived experiences of the participants varied significantly, but the reasons for their persistence were classifiable under five factors, which are described below.

(a) Long-term Participation and Identity as Musicians (the History factor)

An important factor that enabled continued participation in music is that, given that the participants had been involved in music performance for many years prior to arriving in the United States, they self-identified as musicians. The desire not to lose this identity was an important factor that led to continued participation. Participant 4 described the dilemma he faced: “I feel like I am still a musician, but I feel like right now I am not anywhere. I am not African musician, I am not American musician, because America they haven’t get [accepted] me as a musician”. Participant 1, also a seamstress, dressed in a flamboyant style when participating in music because the dress helps her feel like a musician: “if you are a musician you need to dress like one. … You need to have good clothes and your appearance should identify you as a musician”. Participant 3 reiterated that music is integral to his existence: “without music I would not really … be in existence or I would be a different person. … I would be someone negative”. This is echoed by Participant 2 who said almost the same words: “I can say like … I wouldn’t be the way I am if it wasn’t music”.

Participant 6, as a pastor and community leader, has used music performance to express his identities as a musician, Christian, and Asian. Music connects him to his Asian roots: “The Asian person wants to hear Asian music. … The Western they want to learn, hear the Western music”. The union of several identities within the same individual enables him to serve a church congregation with Asian and American roots.

(b) Desirable Effects of Music Performance (the Benefits factor)

The participants continued to perform music because it benefited them. The primary benefit was that music was valued for coping with and managing emotional health problems, a reality of particular importance given the participants’ experiences of trauma and negative emotions. Participant 1 described music as a healthy distraction from distressing thoughts and feelings: “I might have gone through trials but the time I focus my attention to music I tend to forget”. Participants 3 and 4 both described music as “the medicine of trauma”. In fact, participant 3 felt that there cannot be life without music, as “music is life”. He described how music shifted his focus from traumatic memories: “I wasn’t born to see all these dead bodies”. Because music provided an escape route through which to keep negative images of trauma away, it has enabled meaningful living. He added: “people ask me why I am always a happy person … even when I have problems people won’t realize it. … I have used music to make life easier, to accept whatever comes in the way”. Participant 4 agreed: “I take it as … kind of the medicine of trauma because I myself if I feel stressed, I go and just turn on the music and sometimes I just put on the headphones … I focus on the music, then my stress goes slowly”. Participant 5 added more clarity: “It’s like … a painkiller, like drugs.… They are not helping you cure but they are helping you forget that you have pain. … [You] think about something else except that problem”.

Participants often mentioned social connections and building relationships as a separate benefit of music. They saw music as a means of communicating and engaging others. Music was described as the universal language that enabled people of different cultures and languages to understand each other. Participant 6 described music as the “one language”, whereas Participant 5 felt music communicates “without even having to say a word”. This is especially important because most of the participants had limitations in their ability to communicate in English.

(c) Competence, Talent, and Social Recognition (the Experience factor)

The third factor that enabled the continuation of participation in music was talent, skills, and ease of participation. All participants regarded themselves as competent, skilled, and talented. Consequently, less time and effort was needed to prepare their performances. Social recognition of their talent and skills encouraged greater participation in community performances.

Participants 1, 2 and 4 viewed their skills as a gift from God. Participant 4 believed that his God-given talent needed to be shared with others. He described how he discovered his talent: “I discovered it when I was in the church and I tried to teach the choir, write songs for the choir”. He had seen his compositional skills grow while participating in his church and felt the need to share it with other people. Despite the high financial costs associated with music performance, Participant 1 continued music: “You continue because of God”. Participant 2 took a restrictive position, committing to perform only religious music. He saw secular music as disgraceful “world music” and expressed remorse for having performed that type of music in Kampala (Uganda): “I promised to God if I come here, I will never play world music again. … I was playing world music, because … in Uganda we had no jobs”.

Participants 3 and 5 described their songs as covering a range of social, political, and romantic themes. They selected song themes based on context and audience, often featuring romantic song texts that would possibly be viewed by other participants as inappropriate. Outside of the church context, they also had young students learning from them, as well as fans who followed them on YouTubeTM and social media accounts. They appeared to target the youth by featuring popular contemporary African styles such as afropop and rumba.

(d) The Management of Social and Cultural Differences (the Adaptation factor)

The willingness and ability to adapt was another factor contributing to persistence in music performance. The participants identified the music style and language as fundamental areas that needed to change. Participant 3 justified merging American and African styles to satisfy audiences of both backgrounds: “There are styles that Americans want and we also want them. So, it’s upon us the artists to adjust, learn and see the way forward”. He demonstrates the struggle to not lose his own identity in making such changes: “We don’t need to … lose our culture but there is a way we have to marry, you know, to connect two cultures both African and American if we want Americans to be on our side as well”. Participant 1 recognized the need to adapt: “so you have to change your style, and probably start singing in English”. Referring to the merger of languages, Participant 5 stated, “They might enjoy the music and, if they sing along because it’s in English, they get somewhere they hear a word in Swahili and then they force themselves to actually learn that word”.

(e) Locally Organized Refugee Events (the Environment factor)

The large metropolitan area where the participants performed presented barriers, but also opportunities. The State Refugee Resettlement Program, refugee resettlement agencies, nongovernmental organizations, and faith communities provided important avenues for music performances for refugees. These entities organized events in which music and dance from different refugee groups were performed. Taking place seasonally, these events served to bring refugees and other city residents together, facilitating community integration. However, events were infrequent, and participants felt that occasions were too scarce to make a difference. Participant 5 worked for one of the resettlement agencies, but felt they could do more: “Creating more events for the community... would help. … All I need is the publicity”. Participant 3 criticized resettlement agencies for doing too little:

“I know they [refugee agencies] would say they are limited with the resources … but I still disagree with that. ... There are some things that don’t need much resources. ... No one told me ... can we connect you with other musicians or other people? It is me that looked for where to perform. It is me that started looking for a studio. It is me that did. Why? ... What are they there for?”

In addition to community events showcasing refugee music and cultures, Christian churches catering to migrant groups also afforded opportunities, given that the majority of participants engaged in performances during weekly services. These communities used languages from other countries during their services, sometimes interchanging those languages with English. Participant 6, a pastor in one such church, also performed and taught music to youth. Participants 1, 2, 3, and 4 also performed in their respective churches, a context in which their creative talents were greatly valued.

## 4. Discussion

This discussion will emphasize the connections between the aforementioned five factors that contribute to persistence in music performance, and salutogenesis. The factors include long-term participation and identity as musicians (the History factor); desirable effects of music performance (the Benefits factor); competence, talent, and social recognition (the Experience factors); the management of social and cultural differences (the Adaptation factor); and locally organized refugee events (the Environment factor). We now explore how the factors relate to key components of salutogenesis: salutogenic orientation, GRRs/GRDs, and SOC.

First, there is evidence that persistence in music performance has strong links to salutogenetic orientation. It is under the Benefits factor that the artists allude to both pathogenic and salutogenic orientations to health. On the one hand, they indicate that music helps them manage a range of negative emotions resulting from stress or traumatic life experience (pathogenic orientation); but on the other hand, they regard music as their means to integrate into society, promote health, quality of life, and well-being in a new country (salutogenic orientation). They show awareness that health comes from proactive (not just reactive) actions over which they have control. Health is viewed as a continuum rather than a dichotomy, and there is an interest in salutary factors (resources to improve health) and not just risk factors (resources to control or prevent ill health).

Second, a variety of GRRs/GRDs were identified, but the effects of GRRs appear to subdue those of GRDs (e.g., limited finances and lack of English proficiency). Some of the GRRs include musical skills or talent, identity as musicians, commitment to their cultural roots, a variety of coping skills, religion, and social support. By performing music, they use existing skills and express their identities as musicians. The performance of afropop, rumba and other foreign styles of music, along with the use of foreign languages, signifies commitment to their own cultural roots, while the use of music to both reduce negative thoughts or feelings and to foster health and wellbeing shows a deliberate and informed application of resources available to improve health. They composed songs that emphasize religious or philosophical themes related to meaningful living and a hopeful future, and their performance in churches (or similar gatherings organized by other community members and refugee resettlement agencies) signify community involvement and social support.

**Figure 2:**
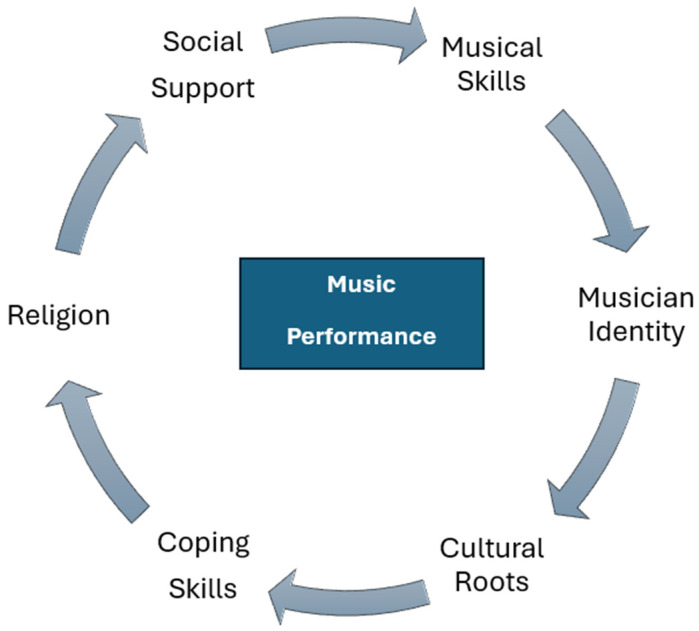
Music performance and general resistance resources.

Music uniquely circumscribed within its performance a variety of GRRs attributable to the life experiences of the participants. Subsequently, as indicated in Figure 2, music is not only an enjoyable creative endeavor, but also the glue that holds a variety of GRRs together. It strengthens the bond between GRRs and augments their expression so that their impact is more pronounced and enduring. Persistence in music is essential in this dynamic process, where maintaining a high SOC (further examined separately below) in changing circumstances can be challenging.

Some identified GRDs included financial challenges, cultural differences, limited English proficiency, and limited audiences. These GRDs could inhibit SOC, but a variety of GRRs, bound together and fortified by persistent music performance, seemed to weaken their effects. For example, in composing songs, participants expressed their religious and philosophical views, which contrasted with more prevalent views that dominated their new American urban setting. Furthermore, because these views were shared with some community members, social bonds of like-minded groups developed, reducing the likelihood of loneliness in a context where isolation and loneliness are likely. Because they see music as having an inherent ability to communicate, even without using words, English language limitations are inhibited and social integration supported.

Although we examine a single activity, music may not be the only type of activity that could unify and fortify GRRs. One can imagine the possibility that a variety of recreational, educational, social, cultural, and creative activities could be a means to unite or fortify multiple GRRs as well. Examples of other activities that have acted in comparable ways include gardening [19], art [20], yoga [21], and religion/spirituality [22]. We support the position that there is a lot about salutogenesis that could be learned from exploratory qualitative studies [3], especially with a focus on specific activities. In this paper, describing persistence in music reveals how a collection of GRRs is jointly shaped by and also gives shape to the performance of music. Understanding this transactional relationship between music and GRRs offers clues as to why SOC may be high for some individuals. That is, it matters what kinds of activity people do as part of everyday living [23]. Understanding how different activities shape SOC could guide important public health policies and other interventions.

The benefit of applying music to promote SOC among immigrants, compared to other activities, lies in several of its known qualities that are not always present in other types of activities. Music is easily carried across borders because it is more malleable and transportable than many activities [24]. It has enormous potential to promote social interaction, facilitate the growth of positive social relationships, and elicit positive emotions and attitudes [25]. Music performance creates opportunities for enjoyment, collaboration, and networking [26,27]. It promotes integration and inclusion, reduces isolation, fosters a sense of identity, and promotes mental and emotional health [14,18,24,28]. Therefore, one can argue that the loss of music as an activity could have profoundly impacted SOC for the participants. On the other hand, interventions that allow similar experiences for immigrants who desire music performance (but are at risk of abandoning participation due to the challenges encountered in the new environment) could promote SOC and support health promotion.

The History factor draws from long-term performance of music and reveals multiple GRRs. These performers engaged in music activities for decades across several countries [14]. Subsequently, they indicate a struggle, an unwillingness to let go of music even when hardships become quite intense, because of a strong desire to retain their identities as musicians and the desire to honor their cultural heritage. The Experience factor represents what Antonovsky called knowledge or intelligence [5]. For the musicians, it meant that being highly competent artists made the activity itself easy to perform and enjoyable. The Experience factor is certainly closely related to the History factor because a long history of performance in any activity naturally leads to greater competence or better skills. However, the two are distinct GRRs, which in Antonovsky’s terms are represented by knowledge (or intelligence) and the ego identity for Experience and History factor respectively.

The Adaptation factor is the GRR that helps the musicians respond positively to their GRDs such as English language problems, financial barriers, differences in music styles (American preferred more than African or Asian), and cultural differences. They show a readiness to compose songs in English, a willingness to change their musical style to suit new audiences, and a willingness to contribute to communities even if they are not remunerated for their services.

SOC is the third and final component of salutogenesis with which music performance is associated. Antonovsky wrote that, confronted with a stressor, an individual with a strong SOC will (1) wish to and be motivated to cope (meaningfulness), believe the challenge is understood (comprehensibility) and believe coping resources are available (manageability) [2]. These three dimensions are strongly represented in the experiences of the participants. They show great motivation to improve health and well-being, believe that they understand the issues related to their own health and the health of other refugees, and see music and religious practices as part of the repertoire of resources to cope. Our study design did not include SOC measures, but one can reasonably expect that the musicians would score high on SOC scales, and the enduring performance of music most probably explains at least part of it.

Music performance functions as an activity that fortifies and unifies general resistance resources (GRRs) and is linked to all fundamental components of salutogenesis. Given that activities have diverse mechanisms of action through which they influence health outcomes, we believe that studying how specific activities are orchestrated in the lives of individuals and how those activities influence GRRs and subsequently SOC may add important perspectives to understanding and applying salutogenesis in practice, research and policy.

One limitation of this study is that it draws from data that were not originally collected to explore links between lived experience and salutogenesis, potentially limiting access to relevant data. Secondly, despite a diverse pool of potential participants, the final sample was more homogeneous than intended. Although potential participants from other cultural backgrounds were present and expressed interest in participating in the study, attempts to arrange interviews were unsuccessful due to conflicting schedules. Participants from more than three countries would have been more representative of the diverse pool of refugee musicians in the area. An additional limitation is that all participants lived and performed in the same geographical context, and refugee musicians in other locations may have differing perspectives.

## 5. Conclusions

This research was carried out to explore the relationship between salutogenesis and musical performance among refugees. Results indicate strong links between music performance and salutogenic orientation toward health, GRRs, and SOC. The participants adopted salutogenic, as well as pathogenic, views on health. Music was a means of integrating and fortifying a variety of GRRs, which led to an enhanced SOC and improved general health and well-being. The acts of music creativity and performance allowed participants to fortify and integrate GRRs allowing them, among other things to: (1) make use of their creative skills, (2) express their identities as musicians, (3) show regard for their own cultures, (4) apply music in coping, (5) express their religious beliefs and values, and (6) find community and social support. The high SOC of the participants appears to be noticeably related to their creativity and musical performance. Considering non-musicians, it is plausible, though it would require additional research to ascertain, that other forms of activity may take a similar role in enhancing SOC. Additionally, studies that test the efficacy of activity-based interventions for building SOC are needed before policy recommendations can be made.

## Figures and Tables

**Table 1:** Study participant demographics. DRC = Democratic Republic of the Congo.

Participant	Country of Origin	Gender	Age	Years in US
1	DRC	Female	48	2
2	DRC	Male	22	2
3	DRC	Male	33	2.5
4	DRC	Male	32	2.5
5	Burundi	Male	29	10
6	Myanmar	Male	47	6

## Data Availability

The raw data supporting the conclusions of this article will be made available by the authors on request.
